# A Perspective Towards More Sustainable Production of Biotechnologically Relevant Enzymes Using DESs

**DOI:** 10.3390/molecules30193915

**Published:** 2025-09-28

**Authors:** Hugo Monteiro, Liane Meneses, Alexandre Paiva, Nuno Galamba, Ana Rita C. Duarte

**Affiliations:** 1 LAQV-REQUIMTE, Faculdade de Ciências e Tecnologia, Universidade Nova de Lisboa, 2829-516 Caparica, Portugal; h.monteiro@campus.fct.unl.pt (H.M.); lp.meneses@fct.unl.pt (L.M.); alexandre.paiva@fct.unl.pt (A.P.); 2 BioISI—Biosystems and Integrative Sciences Institute, Faculty of Sciences of the University of Lisbon, C8, Campo Grande, 1749-016 Lisbon, Portugal; njgalamba@fc.ul.pt

**Keywords:** biotechnological industry, enzymes, deep eutectic solvents, enzyme extraction, intracellular enzymes

## Abstract

The production of enzymes by the biotechnology industry yields high-value products for various sectors, including the pharmaceutical, food, textile, and detergent industries. Although enzymatic production processes are well established, there is a limitation with the purification of intracellular enzymes. These enzymes require extensive downstream separation and purification processes, adding significant labor and costs compared to extracellular enzymes, which are easier to purify. In this work, we conducted a literature review to demonstrate that deep eutectic solvents (DESs) can be a viable alternative, especially as a more sustainable medium for enzyme stabilization and reactions. Additionally, we hypothesize about their potential to extract intracellular enzymes from microorganisms without disrupting their normal functions. Therefore, beyond the current state of the art, we offer a new perspective on a novel approach for producing intracellular enzymes more sustainably and efficiently.

## 1. Biotechnology Industry

The biotechnology industry has grown over the past decades to meet the increasing demand from other sectors, driven by the pressing need to adopt more sustainable practices. Peter E. Carlson’s statement, “the biotechnology industry is not defined by its products, but by the technologies employed in making them”, emphasizes its development over the years.

**Figure 1:**
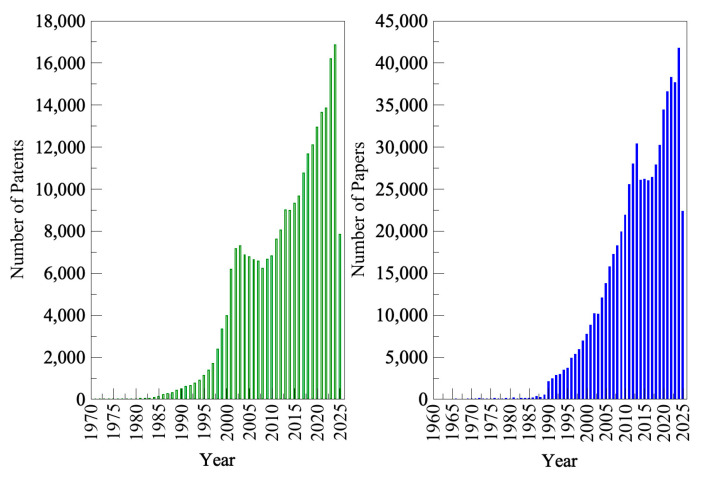
Number of published patents and research papers since 1964 in the field of enzymes and biotechnology. (Search from Espacenet (patents) and Scopus (papers), conducted on 1 July 2025, using the keywords “Enzymes and Biotechnology”).

When the commercial biotechnology industry first emerged [1], the technology was mainly used in the medical, pharmaceutical, and food sectors [2]. However, as new technologies advanced, several other industries began to adopt and explore the benefits of more efficient biotech-based processes. Currently, biotechnology is utilized across diverse fields and is classified as white (industrial biotechnology), blue (marine biotechnology), red (medical biotechnology), or green (agricultural biotechnology), depending on the specific application. White biotechnology involves processing and producing end products and intermediates from renewable resources to generate products for industrial purposes. Blue biotechnology employs biotechnological tools to harness biomolecular processes derived from marine resources. Red biotechnology concentrates on investigating the biological processes of microorganisms to develop new pharmaceuticals and treatments for diseases. Green biotechnology is applied in agriculture to develop technologies that enhance crop productivity and resistance by creating more sustainable fertilizers and biopesticides, thereby benefiting the environment [3]. One of the technologies greatly improved by biotechnology is biocatalysis, which involves accelerating chemical reactions using biocatalysts [4]. Biocatalysts are biological materials, specifically proteins, that can catalyze all reactions involved in cell metabolism. They are capable of catalyzing chemical reactions, reducing reaction times, providing enantioselectivity, and improving production efficiency. Additionally, they contribute to decreased byproduct formation and minimize product inhibition, thereby being regarded as more environmentally friendly alternatives [5]. In recent decades, novel techniques have been developed to produce biocatalysts with enhanced stability and substrate specificity. These include chemical modifications of residues, the addition of protectants to the reaction medium, non-biological approaches to improve stability, and recombinant DNA technology, among others [6]. This has become a powerful technology that addresses specific risks associated with chemical catalysts in various industrial processes, while also being more cost-effective, environmentally friendly, and efficient [4]. These advancements have sparked growing interest from academia and industry in biocatalysis, as evident in the increasing number of publications and patents, as shown in Figure 1. These innovations in biotechnology, especially those related to enzymes, have transformed the market into a billion-dollar industry. In 2021, the demand for industrial enzymes reached USD 6.4 billion [5], with continued growth leading to a value of USD 13.38 billion in 2024 [7], and an estimated rise to USD 15.33 billion in 2025 [7]. By 2029, the market is projected to reach USD 25.88 billion [7].

## 2. Enzymes

Enzymes are the fundamental components of biocatalysis, representing the largest group of proteins essential in various physiological processes such as metabolic pathways, gene expression, and immune responses [8]. They are considered efficient biocatalysts because they can increase reaction rates without altering the equilibrium constant or requiring permanent structural modifications, as conformational changes are often key to catalysis. Additionally, they are generally more effective than traditional chemical catalysts due to their high specificity and selectivity [9]. In nature, enzymes accelerate biochemical reactions within living systems, which can be highly optimized by various conditions. Key factors influencing enzymatic reactions include optimal temperature, pH, water content, substrate and enzyme concentrations, inhibitors, and the accumulation of end products [10]. Enzymes provide high specificity, function under mild conditions, are biodegradable, and generate waste that has minimal environmental impact [11]. This efficiency partly stems from their requirement for fewer raw materials to produce the final product compared to other methods [12]. However, in some cellular processes, such as those involved in producing intracellular enzymes, high energy waste occurs because of the need for extra raw materials for the reaction or purification [13]. A comparison between intracellular enzymes (endoenzymes) and extracellular enzymes (exoenzymes) is shown in Table 1.

The extraction of intracellular enzymes and bioproducts yields a crude matrix that requires further purification steps, such as precipitation, lyophilization, and vacuum drying, among others [14]. Since many enzymes are known, they are classified by the Enzyme Commission (EC) based on the reactions they catalyze, following the EC numbering system. As of 2018, there are seven enzyme classes: oxidoreductases, transferases, hydrolases, lyases, isomerases, ligases, and translocases [8,10,15]. Enzymes are grouped by the type of reaction they facilitate; for example, hydrolases handle hydrolytic reactions, and specifically, proteases cleave peptide bonds. Different enzymes can be obtained through various fermentation processes, depending on the organism producing them and the type of enzyme. In the biotechnology industry, enzyme production is a well-established process, with hydrolases dominating industrial production and applications [16]. Hydrolases can be produced by various organisms through different fermentation processes, as shown in Table 2.

Besides the examples listed in Table 2, there has been increased interest in genetically manipulating enzymes over the past decades to create new (unnatural) enzymes with improved resistance to harsh conditions and to replace toxic materials used in reactions where native enzymes cannot be applied. This leads to improved final products and less resource waste, while enhancing the enzyme conversion rate. Engineering methods, such as directed evolution [35], rational design [36], fusion proteins [37], surface display [38], and the introduction of unnatural amino acids [39], have been employed to enhance enzymes.

### 2.1. Production of Biocatalysts

The biotechnology industry has dedicated significant efforts to optimizing biocatalyst production over the years by examining various factors, such as the microorganism used to produce specific enzymes, the optimal pH for promoting microorganism growth, medium selection—including nutrients that enhance the microorganisms’ growth rate—and enzyme quantities [4]. Currently, industrial enzymes are derived from diverse sources, including fungi (50–60%), bacteria (24–35%), plants (10–15%), and yeast (approximately 4%) [4,39]. The majority of industrial catalysts are obtained from fungal sources due to several advantageous attributes [40]. A primary benefit is that enzymes produced by fungi exhibit a broad spectrum of catalytic activities. Additional important considerations include ease of genetic manipulation, high yields, rapid production utilizing cost-effective media, reproducibility, exponential growth, and ease of optimization [4].

Furthermore, the most critical determinant in selecting the most appropriate microorganism for enzyme production is the target enzyme itself. The principal markets for large-scale enzyme production encompass bioenergy, technical enzymes, food enzymes, animal feed enzymes, and detergents [41]. Proteases and carboxylases, particularly amylases and pectinases, constitute the primary enzymes driving these markets [42].

### 2.2. Advances in Genetic Manipulation to Produce New Enzymes

With advancements in emerging technologies, approximately 90% of industrial enzymes are currently engineered [4], with amylases (nearly 30%) and lipases representing the majority of enzyme production worldwide [43]. As metagenomics has advanced, it provides a powerful tool for extracting gene sets of specific enzymes from microbial sources [44], leading to the development of newly engineered hydrolytic enzymes that aim to improve their inherent properties for industrial applications [4]. Research efforts have focused on enhancing kinetic efficiency [45], thermostability [45,46], pH tolerance [47], enzyme-substrate specificity [45,47], and stereoselectivity [48], as well as often eliminating allosteric regulation [4]. Additionally, genetic engineering techniques are used to clone genes of less abundant enzymes related to microbial growth optimization, enabling their overexpression [45,46,47,48,49]. There is also increased demand for specific enzymes in the medical and pharmaceutical sectors [40]. Advances in genomics [50], proteomics [51], and recombinant DNA technology [52] have greatly supported the discovery and design of new enzymes, replacing more hazardous catalysts and lowering production costs [43].

### 2.3. Production and Purification of Industrial Enzymes

Large-scale enzyme production comprises two principal stages [53], the initial being the selection of the microorganism that synthesizes substantial quantities of the target enzyme, and the subsequent stage involving microbial cultivation, commonly referred to as fermentation or growth [54]. An essential related step consists of selecting a media composition conducive to microbial proliferation. The fermentation process can be executed through three methodologies: (a) batch process [55]; (b) fed-batch process [56]; and (c) continuous process [57]. Batch and fed-batch processes are comparable, distinguished primarily by the timing of medium component addition. In the batch process, all nutrients are introduced at the outset, whereas in the fed-batch process, nutrients are supplied progressively to optimize microbial growth. Conversely, the continuous process sustains a steady state through the continuous input of fresh medium and the simultaneous removal of culture. These methodologies can be implemented via either solid-state fermentation (SSF) [58] or submerged fermentation (SMF) [59], with SSF utilizing moist solid substrates and SMF employing liquid media. Table 3 provides a comparative analysis of these two fermentation processes.

SSF is a straightforward fermentation process on solid substrates that is both cost-effective and eco-friendly. It uses agro-waste nutrients as a medium and requires minimal regulation of process parameters, demonstrating low catabolic repression even when excess substrate is present. Additionally, it produces less effluent waste and reduces foam generation, leading to high enzyme yields with simple purification steps. This method is typically used with fungi [60], as agro-wastes naturally contain the nutrients needed to create suitable growth conditions. However, it has been employed for other microorganisms, such as bacteria and yeast, in recent years.

Alternatively, SMF involves the growth of microorganisms in a liquid substrate with a medium rich in oxygen (depending on the host) and carbon dioxide at optimal pH and temperature. This type of fermentation is typically used to produce bacterial enzymes because it supports bacterial cell growth and enzyme secretion [23]. In SMF, sterilized production media is inoculated with bacterial strains, and fermentation parameters such as aeration, agitation, oxygen, rotation, and pH are maintained over a period of 24 to 72 h, depending on the specific bacterial strain used [23]. SMF is generally not suitable for fungal growth due to issues with viscosity, oxygen, and nutrient transfer in the broth; however, significant progress has been made to address these challenges [61].

**Figure 2:**
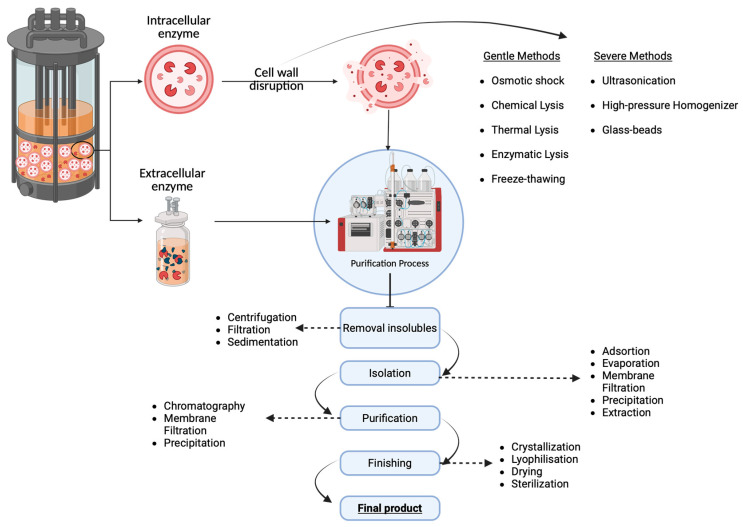
Diagram of the processes used to obtain intracellular and extracellular enzymes.

The SSF offers three primary advantages over SMF: higher volumetric productivity, a higher concentration of products, and reduced effluent generation [62]. During fermentation, microorganisms produce either intracellular or extracellular enzymes, which require different purification methods to obtain the final product for commercialization [63]. After fermentation, the bioproducts are collected, and complex downstream processing is necessary (see Figure 2). This may include the filtration of cell debris [64], purification [64], and the formulation of enzymatic products [65]. The specific steps depend on the type of enzyme produced and its location. Extracellular enzymes undergo a more straightforward downstream process [66], where the enzyme is separated from the medium using techniques such as filtration.

In contrast, intracellular enzymes require a complex purification process because the cell wall must be broken to access the crude matrix, where the bioproduct is dispersed among other intra- and extracellular components [67]. This process adds significant costs to production. To obtain these bioproducts, two methods can be used to induce cell lysis, depending on the cell wall’s resistance: (a) gentle methods [67], and (b) mechanical or harsh methods [67]. Gentle methods are more specific and less damaging to the cell wall, which can sometimes reduce the number of purification steps required. These include approaches like osmotic shock, freeze-thawing, chemical lysis, thermal lysis, and enzymatic lysis [67]. Mechanical methods are more aggressive because they exert considerable stress on the cell wall due to their non-specific nature. Examples include ultrasonication, glass-bead treatment, and high-pressure homogenization [67]. The diagram illustrating the methods used to obtain intracellular and extracellular enzymes is shown in Figure 2.

Once the crude matrix from the cells is obtained, a downstream process is applied to separate organelles and other bioproducts, such as the target enzyme in the mixture. Purification methods rely on the enzymes’ ionic properties, their ability to adsorb onto a resin matrix, and their size [68,69]. The primary goal at this stage is to ensure that the enzymatic product maintains activity and stability and can be released at the application site [70]. Techniques such as chromatography [71], crystallization [72], and electrophoresis [71] are employed to produce the final product. Therefore, enzyme purification is the most costly step in the production process.

### 2.4. Stabilization of Commercial Enzymes

Purification alone does not ensure enzyme stability and activity, since the product must remain stable for delivery to downstream industries. Free enzymes frequently denature when exposed to adverse conditions, such as non-optimal temperatures, pH levels, and other environmental factors [73]. Although the use of enzymes in reactions has made many industrial processes more competitive, their use has been limited by issues such as instability, efficiency, specificity, high production costs, and difficulty in separating and recovering them from the reaction mixture after use [72,74]. One solution to overcome these limitations has been the use of enzyme immobilization [73], which can help increase activity, stability, and reusability. Several efforts have been made to address these concerns, including studying enzyme structure, screening natural enzymes, and immobilizing enzymes for specific applications.

**Figure 3:**
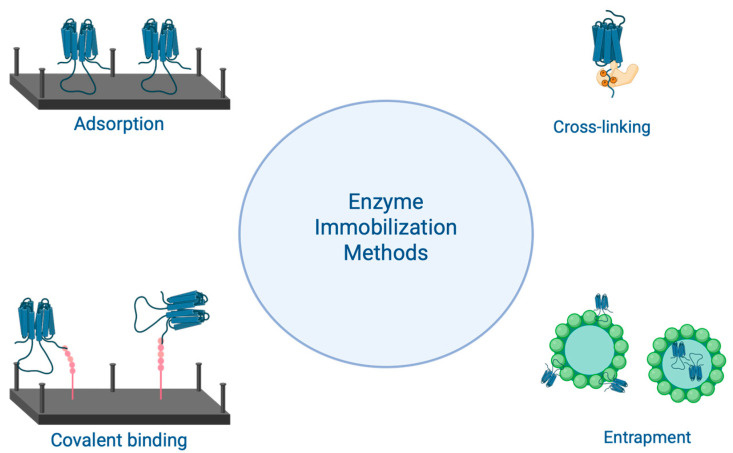
Scheme of the four methods used for enzyme immobilization.

Enzyme immobilization involves trapping the biocatalyst within a matrix or support, such as inert polymers and inorganic materials, thereby enhancing catalytic activity and reusability. This method provides a high investment-to-capacity ratio and improves the purity of the final product [75]. Various approaches can be used for enzyme immobilization, including adsorption, covalent binding, cross-linking, and entrapment [76,77], as shown in Figure 3. These approaches are considered based on factors such as physical strength, regenerability, stability, nonspecific adsorption, microbial contamination, higher specificity, and activity compatibility without inhibiting the final product. The advantages of using immobilized enzymes also include increased enzyme stability, the ability to reuse the same enzyme in multiple reaction cycles, easier recovery and separation of the enzyme from the reaction medium, and the potential to create multi-enzyme reaction processes [77].

A general overview of the methods used for enzyme immobilization is shown in Table 4.

Adsorption is the simplest method of immobilization because it involves forming weak bonds, such as Van der Waals forces and electrostatic and hydrophobic interactions. This approach is considered straightforward and cost-effective, and it does not affect the enzyme. However, it has drawbacks, primarily the leaching of the enzyme, which can occur due to changes in temperature or pH, as the bonds connecting the enzyme to the support are weak [77]. Of the methods mentioned, covalent binding is the most commonly used for enzyme immobilization. In this method, the functional groups of the enzyme and support are linked through covalent bonds, which are more stable. To avoid impairing the enzyme’s activity, non-essential functional groups should be involved in bond formation. Enzyme binding to the support typically involves two main steps: (1) activation of the support surface and (2) coupling of the enzyme to the activated support. Covalent bonding is favored because it offers low enzyme leaching, greater uniformity on the support, and better control over the amount of enzyme immobilized. The main disadvantage of this method is that a high degree of enzyme denaturation can occur during immobilization. Additionally, it requires large reagent volumes to process small amounts of protein and can reduce the enzyme’s activity [77].

Cross-linking is the strongest method used to immobilize enzymes, where enzymes are irreversibly connected through covalent bonds, forming a three-dimensional structure that is not attached to a support matrix. This process utilizes a cross-linking reagent, such as glutaraldehyde, and the most common methods are cross-linking enzyme aggregate (CLEA) and cross-linking enzyme crystal (CLEC). The advantages include minimal enzyme leaching thanks to the strong covalent bonds and the ability to change stabilizing agents to modify the environment and enhance enzyme stability. However, using glutaraldehyde is a significant drawback because it can cause conformational changes that may lead to a loss of activity [77].

Lastly, entrapment is a technique where the enzyme is embedded within a polymer network. During biocatalytic processes, this network facilitates the transfer of reaction components while keeping the enzyme contained within the matrix. Enzymatic entrapment is achieved by mixing the enzyme into a polymer solution that is subsequently polymerized. This method helps minimize enzyme leaching when the pore size is appropriate, enhances stability, and allows customization of the environment for each enzyme. The main drawback of immobilization by entrapment is the limitation in mass transfer caused by the polymer network formed. These polymer networks can be created using various methods, such as photopolymerization, electropolymerization, the silica sol-gel process, or microencapsulation [76,77].

**Figure 4:**
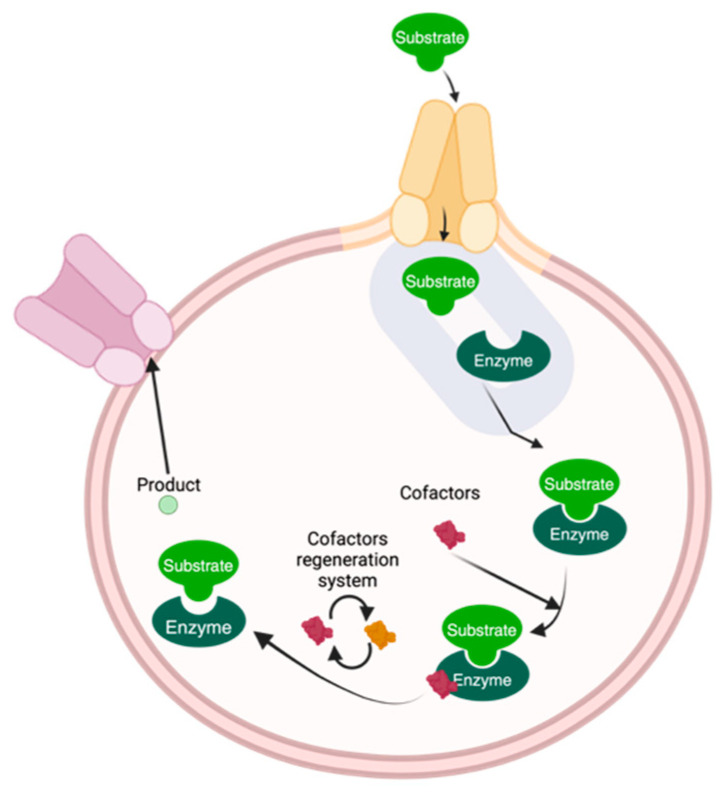
Scheme of whole-cell biocatalysis.

Another strategy is to carry out whole-cell biocatalysis [93] as shown in Figure 4. In this process, enzymes are maintained within intact cells, providing optimal physiological conditions that avoid the need for cofactors in the reaction, facilitate multi-step reactions, and eliminate the requirement for downstream processing. This method is inexpensive compared to other techniques due to the high cost of cofactors usually required. The intracellular enzymes are protected by residual cell wall components, resulting in greater resistance to harsh conditions. Whole-cell biocatalysis is a suitable option for reactions involving hydrophobic substrates [94]. The resistance provided by the cell components enables the use of non-conventional media to dissolve hydrophobic substrates, allowing them to migrate into the cell and produce the final product. The cells can also regenerate cofactors used in the reaction, thereby increasing the yield of the final product with high regio- and stereoselectivity [95]. This method is more environmentally friendly, as it expands the range of solvents that can be used as reaction media, and is economically appealing since microbe-produced cofactors can replace expensive materials [94].

**Figure 5:**
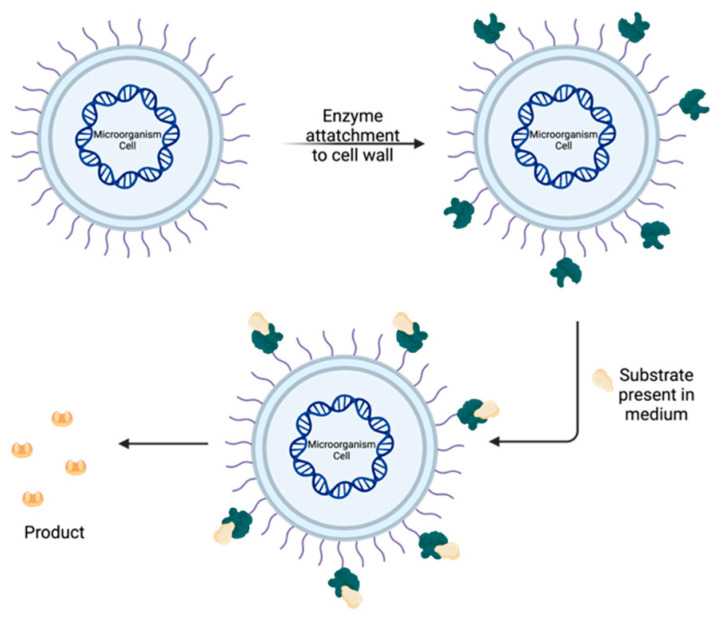
Scheme of cell surface display technology.

Another alternative is cell surface display technology [96], which involves attaching one or multiple proteins to the cell surface, resulting in high productivity through coordinated catalysis, as shown in Figure 5. Unlike whole-cell biocatalysis, the enzymatic reaction occurs outside the cell wall, where the protein/enzyme complex is not limited by the substrate’s ability to diffuse across the wall, thereby allowing easier access to the substrate. This method offers several significant advantages, including the ability to utilize the enzyme in multiple catalytic cycles. Additionally, linking the enzyme to the cell wall reduces proteolytic degradation, leading to higher enzymatic activity. The final product can be easily separated by centrifugation, and both the linkage between the cell and enzyme, as well as the enzyme’s properties, are preserved [38].

To influence the production of commercial enzymes, a more cost-effective process with high productivity and product stability is required. Often, the main limitation to high enzymatic productivity is substrate dissolution, along with the challenge of using environmentally friendly media. To address substrate dissolution, co-solvents and additives like ionic liquids are added to the reactions, but recent concerns have arisen regarding their ecological impact [97]. In recent years, new solutions have been explored to address these limitations, including the use of alternative solvents, where deep eutectic solvents (DESs) have emerged as promising candidates [98].

## 3. Deep Eutectic Solvents

Deep eutectic solvents (DESs) are a class of solvents that can be created by mixing two or more components. When combined, they form a liquid with a melting point lower than that of each component. DESs are typically produced by mixing Lewis or Brønsted acids and bases (hydrogen bond acceptor (HBA); hydrogen bond donor (HBD)) and may include various anionic or cationic species [99]. In recent years, different classes and subclasses of DESs have been identified. One classification is based on the nature of the compounds used to make the DESs, such as natural deep eutectic solvents (NADESs) [100]. NADESs are formed by combining natural components, usually resulting in systems that are low in toxicity, highly biodegradable, and more biocompatible [101]. DESs have a wide range of potential applications across industries, including pharmaceuticals, cosmetics, biotechnology, textiles, and food, and have been thoroughly reviewed in the literature [99,102]. A main drawback of these solvents is their high viscosity. Although high viscosity can limit their use in some applications, adding a small amount of water or conducting processes at higher temperatures can significantly reduce viscosity, often by several orders of magnitude [103,104].

## 4. DESs and Living Systems

### 4.1. Studies of Deep Eutectic Solvents and Enzymes

**Figure 6:**
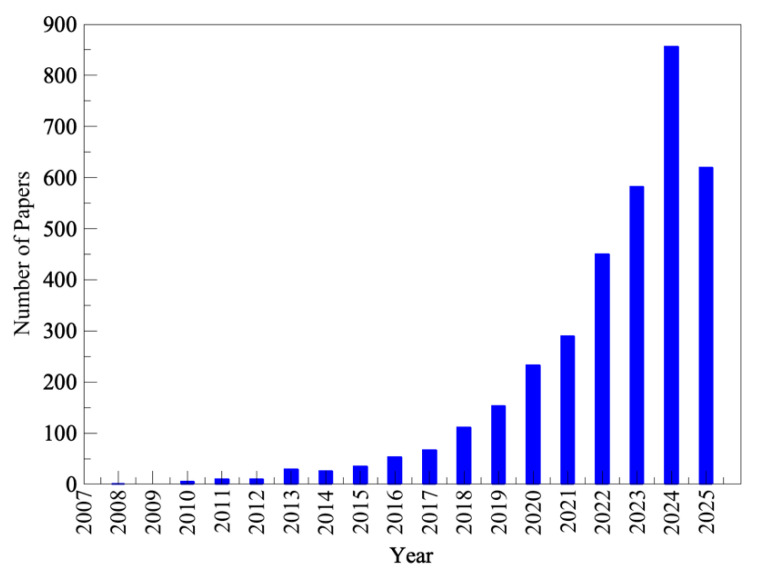
Number of publications since 2008 in enzymes and deep eutectic solvents. (Search conducted using Scopus (papers) on 1 July 2025, with the keywords “Enzymes and Deep Eutectic Solvents.”).

In recent years, growing interest has emerged in the potential use of DESs as a sustainable medium for enzymatic reactions (Figure 6). Verpoorte et al. [105] hypothesized in 2011 that NADESs could be “the third liquid phase in organisms in which certain biosynthetic steps or storage of products may occur.” This relates to the fact that many NADESs are formed by osmolytes produced by living organisms, creating a crowding environment that protects biomolecules under stress conditions (e.g., dehydration, heating, cooling [106]). Since then, there has been increasing exploration of these solvents as reaction media, co-solvents, or even solvents and substrates for enzymatic chemical reactions [107]. The use of DESs in biocatalysis offers several additional advantages, including improved activity and thermostability, enhanced substrate solubility, and increased selectivity [108].

The specific behavior of enzymes in NADESs depends on each system’s unique features, such as viscosity, water content, water activity, and acidity. This makes it crucial to perform case-by-case studies since different combinations will exhibit varied qualities [109]. Recently, Arnodo et al. reviewed numerous reports of biocatalysis in DESs and NADESs, organizing them by reaction type to produce a summary with over 70 examples of more sustainable alternatives to traditional reactions. In addition to being used as solvents or substrates in reactions [110], NADESs have also been employed to stabilize enzymes, enhance their thermostability, enable immobilization, and help in the preparation of nanoparticles.

From this perspective, we review several studies that explore DESs with different classes of enzymes and reactions, such as esterification, hydrolysis, and alcoholysis (frequently known as transesterification). In these reports, enzymatic activity, thermostability, affinity, and kinetics are assessed and compared in neat DESs and DES–water mixtures with traditional methods used over the past few decades [111,112].

Molina et al. [113] investigated the use of choline chloride-based DES as a reaction medium and co-solvent for converting starch or maltotriose into alkyl glucosides with α-amylase. This enzyme catalyzes reactions such as hydrolysis and alcoholysis. The authors examined the potential of DESs as solvent media, demonstrating that small amounts of water are necessary to maintain the enzyme’s activity and stability in choline chloride-based DESs. They also found that, although the enzyme was nearly fully deactivated in pure DESs due to their high viscosity, its performance improved when DESs were used as a co-solvent. Adding water, especially to reline (choline chloride:urea (1:2)), increased the reaction’s selectivity for producing alkyl glucosides. Furthermore, increasing DES concentration led to a decrease in both hydrolysis and alcoholysis reactions, especially hydrolysis, suggesting that DESs can be used for selective reactions. The enzyme’s thermostability and structure were also studied, revealing that at 60 °C, the ratio of alcoholysis to hydrolysis increased without damaging the enzyme’s conformation. Additionally, the alcoholysis yield with the optimal DES as a co-solvent was 20% higher than previously reported using an aqueous buffer.

In another study, Cao et al. [114] explored the activity of the enzyme β-glucosidase and Candida antarctica lipase B in nearly anhydrous DESs. The authors used different HBA-based DESs and HBD-based DESs, specifically, choline chloride-based, urea-based, and glycerol-based DESs with hydrophobic and hydrophilic properties, to assess the biocompatibility and thermostability of the enzymes. A correlation was identified between biocompatibility and DES hydrophobicity for the stabilization of enzymes. The hydrophobic DESs exhibited lower biocompatibility compared to the hydrophilic DESs, and acid-hydrophilic DESs could cause irreversible enzyme inactivation. Another aspect examined was the effect of different HBAs (choline chloride, tetraethylammonium bromide, menthol, and decanoic acid) and HBDs (glycerol and decanoic acid) on enzyme performance. These experiments indicated that the use of acidic HBDs negatively impacted enzyme biocompatibility and thermal stability due to their acidity and polarity. Conversely, a different trend was observed with bases as HBDs. Interestingly, contrasting results emerged when analyzing the effects of HBAs, where hydrogen bond acidity and polarity were found to influence enzyme biocompatibility and thermal stability positively.

Toledo et al. [115] studied how aqueous solutions of 16 cholinium- and betaine-based DESs at different molar ratios, along with their individual HBA and HBD components in water, influence the stability and activity of laccase. They discovered that these DESs kept enzyme activity stable at 60 °C after two days of incubation. Additionally, the DESs significantly enhanced thermal stability. At −80 °C and over 20 days, laccase maintained activity levels ranging from 130% to 200% of the initial activity. Even after this period, storing laccase in DESs resulted in higher activity compared to the control. In this research, choline dihydrogen citrate:xylitol (ChDHC:Xyl) (2:1) at 25 wt% was identified as a promising DES for enzyme storage. The authors concluded that while the ChCl-based DES reduced enzyme activity, replacing the chloride anion in cholinium salts with dihydrogen citrate increased laccase activity, thereby improving its performance in oxidative reactions.

Another example is the study by Gonzalo et al. [116] on the use of NADESs as co-solvents to evaluate the performance of 5-hydroxymethylfurfural oxidase (HMFO) in producing furan-2,5-dicarboxylic acid (FDCA). The authors successfully carried out oxidative reactions using glucose- and fructose-based DESs. High conversion rates were achieved for synthesizing oxidized compounds, and NADESs improved the solubility of the substrates involved in the reaction, offering strong stabilization for HMFO. Additionally, NADESs, specifically, Glu:fru:H_2_O (glucose:fructose:water) in a 1:1:6 ratio, increased the thermostability of HMFO, indicating the potential for more selective FDCA production at higher ratios using DESs.

Juneidi et al. [117] studied the use of DESs as co-solvents in water-based solutions and as the main solvent for stabilizing and enhancing the activity of Burkholderia cepacia lipase (BCL). The authors compared the enzyme’s performance in methanol, ionic liquids (ILs), and phosphate buffer. Notably, the enzyme was nearly deactivated in pure DESs, but adding a small amount of water (4% *v*/*v*) increased enzymatic activity and improved kinetics. Compared to other solvent media, the DES:water (4% *v*/*v*) mixture increased enzymatic activity by up to 2.6 times over the buffer, and 1.5 and 14 times over ILs and methanol, respectively. Choline chloride:ethylene glycol in the presence of a buffer medium (40% *v*/*v*) enhanced lipase activity by up to 230% compared to pure DESs. Impressively, the authors found that enzyme activity is influenced more by the DES itself than its individual components. Regarding enzyme thermostability, after incubation at 60 °C, 50% of the initial activity was retained in the DESs after 7 days of incubation.

Aqueous solutions of choline chloride-based DESs were studied as co-solvents by Kim et al. [118] to examine their effect on the activity and stability of Candida rugosa lipase. This research showed that selecting specific HBDs can improve enzyme activity and stability. For example, when glycerol was used as an HBD, the lipase’s half-life increased by 9.2 times compared to buffer at 40 °C. Conversely, using HBDs like formamide did not enhance the lipase’s activity and stability. In their conclusion, Kim et al. established a direct link between the enzyme’s thermal and storage stability and the type of acidic hydrogen bond used to form the DES.

To explore new strategies for synthesizing chiral drugs from hydrophobic substrates, Fredes et al. [119] examined the effect of mixtures of choline chloride:urea (ChCl:U) in 50% *v*/*v* phosphate buffer with Novozym 435. The authors showed that ChCl:U buffer could enhance enzyme selectivity for synthesizing chiral drugs by 16% compared to pure buffer. Using a high substrate concentration in DES mixtures, they achieved 99% purity of enantiomeric products, along with improved enzyme stability at pH 7 and 40 °C, after 27 h. These promising results indicate that DESs are a viable approach for synthesizing specific enantiomers.

All the abovementioned works examined enzymatic activity and thermostability using DESs. Recently, Hümmer et al. [120] explored DESs as both solvent and reaction medium simultaneously for esterification reactions catalyzed by lipases. They investigated DESs made from (DL)-menthol and fatty acids, and the authors observed the synthesis of (DL)-menthol fatty acid esters by *Candida rugosa* lipase in pure DESs. Usually, one common strategy in esterification reactions is to remove water to shift the equilibrium toward ester formation. However, the authors noted that the presence of water (10 wt%) actually increased the esterification of fatty acids. These impressive results suggest that DESs can serve more than one function in enzyme reactions, enabling the replacement of harsher solvents and reducing dependence on raw materials, which can make the process more cost-effective.

In the same theme, Zeng et al. [121] explored enzymatic selective esterification using glycerol-based (HBD) DESs with quaternary ammonium compounds (e.g., ChCl, benzalkonium chloride, cetalkonium chloride) as both substrate and solvent. Their results showed that the choice of the quaternary ammonium compound directly influenced the chemical reaction characteristics of glycerol and lipase and, therefore, enzyme selectivity. The authors reported that ChCl had a significant impact on the esterification of 1,3-DAG catalyzed by Novozym 435 (42.9 mol% in 1 h).

Using similar principles, Guajardo et al. [122] demonstrated that DES–water mixtures composed of choline chloride and glycerol, used for the selective enzymatic synthesis (from lipases) of α-MBG (α-monobenzoate glycerol) with glycerol as the substrate, resulted in a high conversion rate (99%) when the DES–water mixture served as both substrate and solvent. Following the initial batch, 37% of the enzyme activity was depleted, with no additional loss observed in subsequent reaction cycles conducted by the authors.

Another example of the potential of DESs is the demonstrated usage of these solvents in the presence of cells by Mao et al. [123]. The authors investigated the use of choline-based DESs as co-solvents to enhance the bioconversion efficiency of cortisone acetate (CA) to prednisone acetate (PA), using immobilized whole cells from *Arthrobacter simplex*. The bioconversion of CA to PA exceeded 98% with reline as a co-solvent, indicating the potential of DESs for bio-dehydrogenation reactions in industry.

More recently, Cao et al. [124] published a study examining the release of intracellular enzymes using hydrophobic DESs. The authors compared methods used in the biotechnology industry, specifically, sonification and DESs, to stimulate the release of phospholipase D (PLD) from recombinant *Escherichia coli*. The study demonstrated that intracellular components (i.e., metabolites) could be extracted without cell disruption, achieving a higher yield (114.58%) compared to sonification, along with a nearly twofold increase in the specific activity of PLD. A summary of the studies discussed on using DES with enzymes is provided in Table 5.

### 4.2. DESs and Microorganism Lines

Along with the effects of DESs on enzymes, several studies [125,126] have explored the toxicological impact of this class of solvents on different cells and organisms.

For instance, Juneidi et al. [127] investigated the toxicological effects of choline chloride-based DESs on four different fungal strains by examining growth inhibition in the presence of DESs and the lethal concentration at 50%. The authors found that DESs are less toxic than the original compounds used to create them, and acidic DESs showed low inhibition of the growth of the evaluated strains.

Rodríguez-Juan et al. [128] explored the toxicity of DESs to phytopathogenic bacteria and wine yeasts. In comparison with conventional solvents such as DMSO and glycerol, NADESs composed of sugars glucose:fructose:sucrose (Glu:Fru:Suc-1:1:1; 2:3.6:1) show low toxicity toward yeast and bacterial cells. DESs composed of betaine:sucrose (2:1) and choline chloride:sucrose (1:2) exhibited low toxicity toward yeast cells. Choline chloride:sucrose (1:2) and choline chloride:xylitol (2:1) were less toxic to bacterial cells than conventional solvents. On the other hand, DES comprising betaine:sucrose (4:1) showed higher toxicity, suggesting it could be used against bacterial cells as an antimicrobial.

Yang et al. [129] investigated whole-cell biocatalysis using *Lysinibacillus fusiformis* CGMCC1347 cells to convert isoeugenol into vanillin with DESs and NADESs as co-solvents (20% (*v*/*v*)). The authors selected ChCl and ChAc as HBA, and organic acids, alcohols, and sugars as HBD to form DESs. They assessed bioconversion effectiveness, cell viability, interactions with DESs, and the recyclability of the immobilized strain. The study found that more cells survived in ChCl-based DESs, and more intracellular components were released with ChAc-based DESs, suggesting that disrupting the cell wall improves solvent access to enzymes, thus increasing conversion yield. Moreover, the authors observed higher yields when testing the catalytic activity of immobilized cells with certain DESs. Specifically, ChCl:Gal (5:2) (choline chloride: galactose), ChCl:PG (1:1) (choline chloride: 1,2-propylene glycol), and ChCl: EG (1:1) (choline chloride: ethylene glycol) achieved yields of 181,4%, 151,7%, and 145%, respectively, which are higher than yields in pure water. The immobilized cells demonstrated excellent operational stability, maintaining activity for at least 13 cycles.

**Figure 7:**
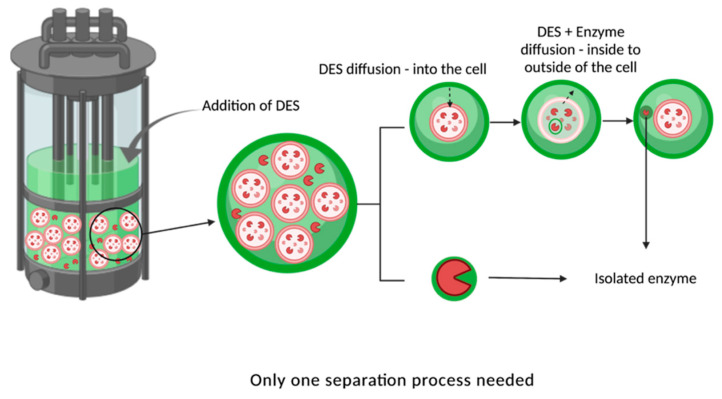
Proposed scheme for applying DES to intracellular enzymes extraction.

Based on the knowledge gained so far, various approaches and applications of DESs have demonstrated remarkable results. Depending on the HBAs and HBDs used to form the DESs, it is possible to develop new hypotheses for their use in the biotechnological industry, ranging from extending storage time to enhancing enzyme affinity and selectivity for specific final products or substrates. With this in mind, we propose a novel potential application of DESs in biotechnology: utilizing DESs as a solvent, co-solvent, or component of nutrient medium for cell growth in a bioreactor, with minimal impact on microorganisms. Since these compounds are naturally produced by cells [130], it is expected that DESs might support normal microbial growth and bioproduct formation. If this hypothesis is correct, and based on existing studies as well as previous research on osmolytes [131,132] not discussed here, it can be theorized that DES components could diffuse into and out of cells without damaging the cell wall [124] and could selectively extract intracellular enzymes into the external medium. This process could significantly reduce the need for traditional purification methods, such as cell wall disruption, and decrease downstream processing costs and enzyme production expenses. A diagram of the proposed hypothesis can be seen in Figure 7.

Despite the theoretically remarkable potential application of this hypothesis, some limitations are present in the research, particularly regarding the selection of DESs that can meet all the necessary parameters for enzymatic activity, microorganisms, and bioreactor operation. Thus, while specific DESs might improve the enzymatic activity of a particular enzyme, in other cases, they may have the opposite effect or even cause protein denaturation. Furthermore, some DESs might disrupt the cell wall, while others will diffuse into the cell. Among the latter, some may be capable of extracting intracellular enzymes, whereas others may not interact with the enzymes present inside the cell. Another drawback of this hypothesis is the scalability of DESs, as some DESs that appear stable in small volumes may not remain stable when the sample volume increases. Nevertheless, given the many variables involved in designing DESs, whether as solvents or co-solvents, it should be possible, in our view, to custom-design biocompatible DESs with low toxicity that can overcome these challenges.

## 5. Conclusions

In recent decades, the biotechnology industry has investigated novel methodologies for enzyme production, including the optimal selection of organisms, growth conditions, and the limitations associated with separation techniques, among other approaches, in order to generate non-natural enzymes that fulfill industry requirements. Nevertheless, specific challenges persist, with intracellular enzymes representing a primary concern. In this work, we examine a new class of environmentally friendly solvents (deep eutectic solvents) and their application in the processing of enzymes and microorganisms. The literature from various researchers over recent years provides compelling evidence supporting the hypothesis that DESs may enhance the extraction of intracellular enzymes without detrimental effects on cell cultures, thereby facilitating enzyme production through a continuous process and obviating the need for multiple downstream steps. Consequently, some DESs with low toxicity towards cell cultures indicate that these solvents could be incorporated into the culture medium within bioreactors. Furthermore, several DESs have demonstrated their capacity to preserve enzyme structure and activity, and even to augment thermal stability. Additionally, a study has shown that DESs can extract intracellular enzymes with high yield, further reinforcing the potential of DESs to enable more efficient extraction of intracellular enzymes.

## Figures and Tables

**Table 1:** Comparison between intracellular and extracellular enzymes.

	Endoenzymes	Exoenzymes
Production	Inside cells	Inside cells
Function location	Inside cells	Outside cells
Function	Facilitate biochemical reactions within the cell	Breakdown of the extremity of the polymer to form monomers one by one
Digestion	Inside the cell	Outside the cell

**Table 2:** Types of hydrolases produced by the biotechnology industry through two fermentation methods, submerged fermentation (SMF) and solid-state fermentation (SSF), and their functions in cells.

Enzymes	Function	Fermentation ^(a)^	Application Field	Intracellular Enzyme	Extracellular Production
Proteases (Hydrolases, EC 3)	Hydrolysis of proteins	SMF [17] SSF ** [17]	Detergent; Pharmaceutical; Food	Protease Lon [18] e.g., *Pseudomonas* *aeruginosa*	Cysteine Proteases [19] e.g., *Bacteria*, *archaea*, and fungi
Cellulases * (Hydrolases, EC 3)	Conversion of cellulose from plants into sugars	SMF [20] SSF [20]	Textile	Cellulases [21] e.g., *Aspergillus oryzae*	Cellulase [22] e.g., *Trichoderma reesei*
Xylanases (Hydrolases, EC 3)	Hydrolysis of hemicellulose	SMF [23] SSF [24]	Food; Pharmaceutical; Textiles; Paper	Xylanases I and II [25] e.g., *Penicillium sclerotiorum*	Xylanase IXT6 [26] e.g., *Geobacillus stearothermophilus*
Lipases (Hydrolases, EC 3)	Conversion of lipids and fats into fatty acids, glycerol, and other molecules	SMF [27] SSF [27]	Food; Detergent; Pharmaceutical; Leather; Textile; Cosmetic; Paper	Hormone-sensitive Lipase [28] e.g., humans and mouse	Triacylglycerol acyl hydrolase [29] e.g., *Bacillus subtilis*
Amylases * (Hydrolases, EC 3)	Hydrolysis of complex carbohydrates into sugars	SMF [30] SSF [30]	Food; Fermentation; Textile; Paper; Detergent; Pharmaceutical	α-amylase [31] e.g., *Paenibacillus* sp.	α-amylase [32] e.g., *Pseudogymnoascus* sp.
Phytases * (Hydrolases, EC 3)	Converts phytate into phosphorus	SMF [33]	Food	Phytases [34] e.g., *Lactobacillus plantarum*	Phytases [34] e.g., *Lactobacillus plantarum*

* Same structural characteristics and functions are found in intra- and extracellular space environments. ** Preferred method: ^(a)^ Fermentation process depends on the type of microorganism used.

**Table 3:** Comparison between fermentation processes used in industry: solid-state fermentation (SSF) and submerged fermentation (SMF).

Fermentation Process	Solid State Fermentation (SSF)	Submerged Fermentation (SMF)
Microorganism preference	Fungi	Bacteria and yeast
Medium composition	Agro-waste nutrients	Liquid substrate (e.g., molasses and broths) rich in oxygen and carbon dioxide
Regulation	Low	High (medium, pH, temperature)
Costs	Low costs due to the usage of agro-waste nutrients	High cost due to the required media components
Effluent	Less effluent waste	Higher effluent waste
Enzyme production	High volumetric production	Low volumetric production

**Table 4:** Overview of methods used for enzyme immobilization.

Method	Immobilization	Molecules Used for Immobilization	Advantages
Adsorption	Hydrophobic interaction and salt linkages [78]	Coconut fibers [79]; microcrystalline cellulose [80]; micro/mesoporous with thiol functionalized [81]	Enzyme shield from aggregation, proteolyss, and interaction with hydrophobic surfaces [78]
Covalent Binding	Association between side chain amino acids (arginine, aspartic acid, histidine) with functional groups [75]	Functional groups: imidazole, indolyl, and phenolic hydroxyl [75]	Higher specific activity and stability [82]; increase in half-life and thermal stability [83]
Cross-Linking	Two distinct methods: 1-Enzyme conjugated with a unit with high affinity to the matrix [84] 2-Matrix precoupled to an affinity ligand for target enzyme [84]	Alkali stable chitosan-coated porous silica beads [85]; Agarose-linked multilayered concanavalin [86]	Can be used for simultaneous enzyme purification through cross-linked enzyme aggregates (CLEAs) and cross-linking enzyme crystals (CLECs) [87]; Ability to harbor higher amounts of enzymes, increasing the stability and efficiency [85,86]
Entrapment	Cage by covalent or non-covalent bonds within gels or fibers [88]	Encapsulation with alginate-gelatin-calcium hybrids Nanostructured supports such as electro spun nanofibers and pristine materials [89]; Entrapment by mesoporous silica [83]; Sol-gel matrices [90]	High thermostability [91], high affinity, and enhancement of activity [92]

**Table 5:** Summary of the impact of DESs on the enzymes examined in the studies and their benefits.

Enzyme	Study	Advantages
α-amylase	DES used as a reaction medium and co-solvent for converting starch or maltotriose into alkyl glucosides	DESs showed that can be used for selective reactions
β-glucosidase and Candida antarctica lipase B	DES used to explore biocompatibility and thermostability of the enzymes	Increase enzyme biocompatibility and thermal stability
Laccase	The influence of DES in the stability and activity to increase storage time	Enzyme activity stable at 60 °C after two days of incubation; At −80 °C and over 20 days in storage, activity levels increased
5-hydroxymethylfurfural oxidase (HMFO)	DES used for the production of furan-2,5-dicarboxylic acid (FDCA)	Strong stabilization for HMFO and increased the thermostability
Burkholderia cepacia lipase (BCL)	Evaluation of the stability, activity, and thermostability	Enzymatic activity increased (by up to 2.6 times over the buffer) and improved kinetics
Candida rugosa lipase	Effect on the activity and stability	Improve enzyme activity and stability; half-life increased by 9.2 times compared to buffer
Novozym 435	Synthesis of chiral drugs from hydrophobic substrates	Enhance enzyme selectivity by 16%; 99% purity of enantiomeric products
Candida rugosa lipase	Esterification reactions	Increased the esterification of fatty acids
Novozym 435	Enzymatic selective esterification	Increased the esterification of 1,3-DAG
Immobilized whole cells from *Arthrobacter simplex*	Bioconversion efficiency of cortisone acetate (CA) to prednisone acetate (PA)	High potential of DESs for bio-dehydrogenation reactions
Lipases	Selective enzymatic synthesis of α-MBG (α-monobenzoate glycerol)	High conversion rate (99%)
Phospholipase D (PLD)	Release of intracellular enzymes using hydrophobic DESs	Intracellular components could be extracted without cell disruption
